# Association Between Bradyarrhythmia Requiring Permanent Pacemaker Implantation and Epicardial Adipose Tissue in Elderly Patients

**DOI:** 10.1002/joa3.70340

**Published:** 2026-04-16

**Authors:** Shunsuke Tomomori, Keita Kimura, Naoya Hironobe, Kiho Itakura, Youji Urabe, Toshiharu Oka, Naoya Mitsuba, Yukihiro Fukuda, Hironori Ueda, Yukiko Nakano

**Affiliations:** ^1^ Department of Cardiology Hiroshima Prefectural Hospital Hiroshima Japan; ^2^ Department of Cardiovascular Medicine Hiroshima University Graduate School of Biomedical and Health Sciences Hiroshima Japan

**Keywords:** bradyarrhythmia, epicardial adipose tissue, pacemaker

## Abstract

**Background:**

Aging is one of the most significant risk factors for sinus node (SN) and atrioventricular node (AVN) dysfunction. Epicardial adipose tissue (EAT) is reported to promote myocardial fibrosis and influence intracardiac conduction through the release of inflammatory cytokines and direct tissue infiltration. The SN and AVN are in contact with EAT, which may affect the SN function and AVN conduction. The purpose of this study was to examine the association between bradyarrhythmia and EAT.

**Methods:**

We enrolled 103 patients with bradyarrhythmia requiring permanent pacemaker (PM) implantations and 105 patients who underwent catheter ablation of paroxysmal supraventricular tachycardia in our hospital as a control (all patients were over 65 years old) and analyzed the preoperative echocardiograms retrospectively and measured the EAT thickness. We compared the EAT thickness between the PM group and control.

**Results:**

There were 38 (36.9%) PM group patients with sick sinus syndrome and 65 (63.1%) with advanced atrioventricular block. The EAT was significantly thicker in the PM group than control (PM group 4.6 ± 1.2 mm vs. control 3.6 ± 1.3 mm, *p* < 0.0001). The EAT was also significantly thicker in the PM group than control (PM group 4.5 ± 1.2 mm vs. control 3.6 ± 1.3 mm, *p* = 0.0017) after propensity score matching (age, gender, hypertension, history of heart failure, left atrial diameter, and estimated glomerular filtration rate).

**Conclusions:**

The EAT thickness was associated with bradyarrhythmia requiring permanent PM implantation and may become a risk factor for SN and AVN dysfunction in elderly people.

## Graphical abstract

We examined the association between the epicardial adipose tissue (EAT) thickness and bradyarrhythmia requiring permanent pacemaker (PM) implantation in elderly people. The EAT was significantly thicker in the PM group than in the control. The EAT may be associated with sinus node and atrioventricular node dysfunction.

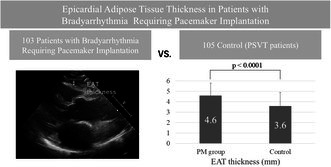

## 1 Introduction

Bradyarrhythmia, such as sinus node (SN) dysfunction and atrioventricular node (AVN) dysfunction, is a common reason for permanent pacemaker (PM) implantation. A greater body mass index, hypertension, and diabetes are risk factors causing SN and AVN dysfunction, but aging is one of the most significant risk factors for SN and AVN dysfunction [1, 2, 3]. It has been reported that the prevalence of atrioventricular block (AVB) dramatically increases from age 53–57 years (men: 1.15%; women: 0.48%) and then reaches a peak at an age ≥ 78 years (men: 7.49%; women: 2.97%) [4]. Epicardial adipose tissue (EAT) has been reported to promote myocardial fibrosis and influence intracardiac conduction through the release of inflammatory cytokines and direct tissue infiltration [5, 6, 7], and has also been associated with atrial arrhythmias [8]. The SN and AVN are in contact with EAT [9, 10], which may affect the SN function and AVN conduction, but there are few reports about the association between EAT and SN and AVN dysfunction. In this study, we examined the association between the amount of EAT and bradyarrhythmia requiring permanent PM implantations in elderly people.

## 2 Methods

### 2.1 Participants

**FIGURE 1:**
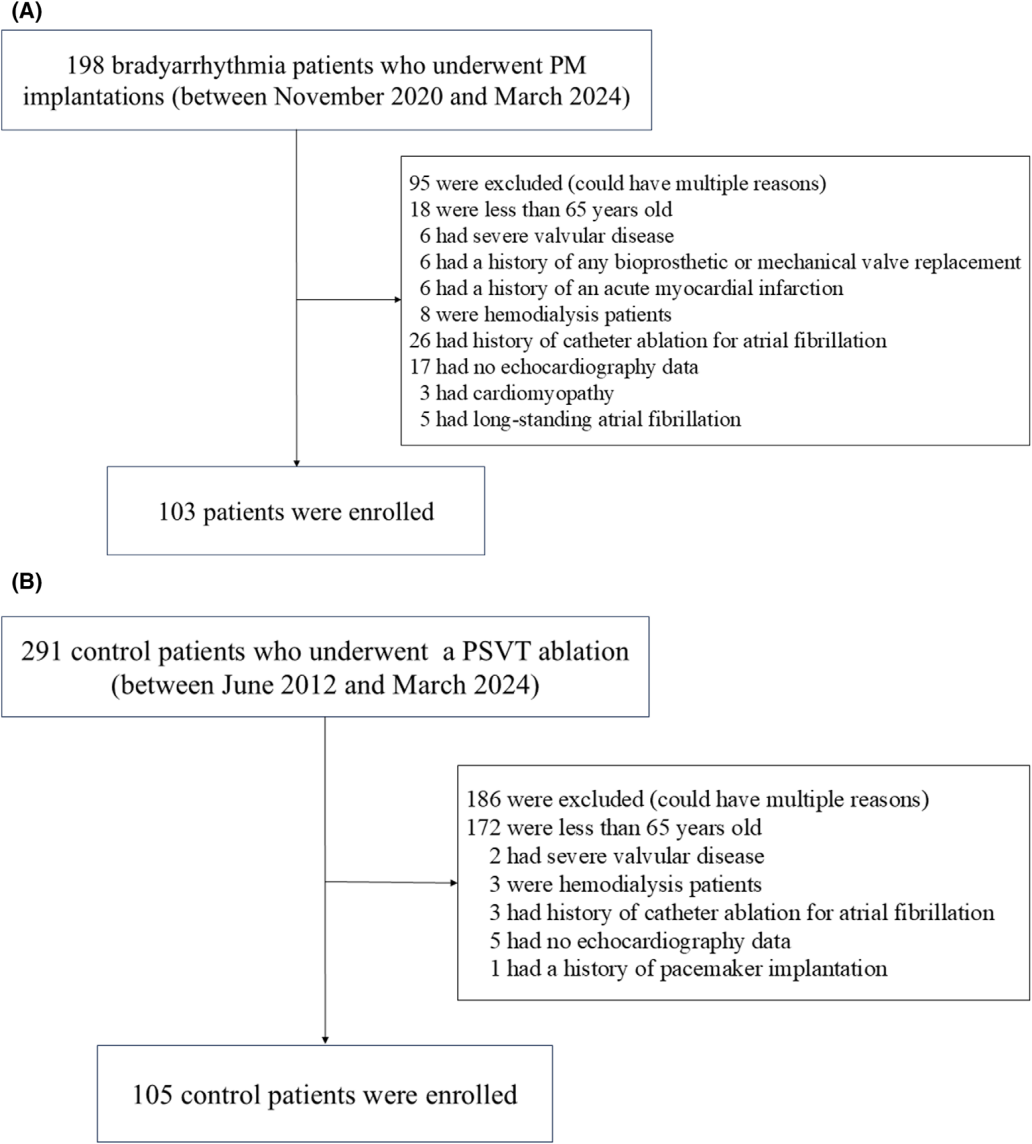
Flow chart detailing the study design in the PM group (A) and control (B).

We screened 198 consecutive bradyarrhythmia patients who underwent permanent PM implantation at Hiroshima Prefectural Hospital from November 2020 to March 2024, and excluded those less than 65 years old (*n* = 18), and those with severe valvular disease (*n* = 6), a history of any bioprosthetic or mechanical valve replacement (*n* = 6), a history of an acute myocardial infarction (*n* = 6), hemodialysis (*n* = 8), a history of catheter ablation of atrial fibrillation (AF) (*n* = 26), those who had no echocardiography data (*n* = 17), those with cardiomyopathy (hypertrophic cardiomyopathy, cardiac sarcoidosis, and cardiac amyloidosis) (*n* = 3), and those with long‐standing (defined as lasting more than one year) AF (*n* = 5) (Figure 1A). We also screened 291 control patients who underwent catheter ablation of paroxysmal supraventricular tachycardia (PSVT) at Hiroshima Prefectural Hospital from June 2012 to March 2024, and excluded those less than 65 years old (*n* = 172) and those with severe valvular disease (*n* = 2), hemodialysis (*n* = 3), a history of catheter ablation of atrial fibrillation (*n* = 3), no echocardiography data (*n* = 5), and a history of pacemaker implantation (*n* = 1) (Figure 1B). The Institutional Ethics Committee of Hiroshima Prefectural Hospital approved all procedures.

### 2.2 Echocardiogram Measurements and the EAT Thickness

**FIGURE 2:**
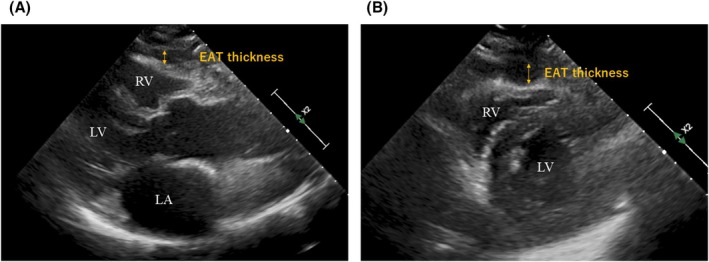
Typical examples of the echocardiographic measurement of the EAT thickness. We analyzed the preoperative echocardiograms retrospectively and measured the EAT thickness twice in the long (A) and short‐axis views (B).

Transthoracic echocardiography was performed at our institution using a commercially available system (Vivid E9, GE Healthcare, EPIQ7C, Philips, Aplio i700, Canon) before the PM implantation or catheter ablation. We analyzed the preoperative echocardiograms retrospectively and measured the EAT thickness twice in the long and short‐axis images, as previously reported [11]. The EAT thickness was measured by two blinded observers with extensive echocardiographic experience (Figure 2A,B). The inter‐observer correlation coefficient was 0.97. We compared the mean values between the PM group and the control.

### 2.3 Statistical Analysis

Normally distributed continuous variables are presented as the mean ± standard deviation. Group differences among the continuous variable were analyzed using a two‐sided *t*‐test or the nonparametric Mann–Whitney *U* test and among the nominal variable using a chi‐squared test. A *p*‐value of < 0.05 was considered statistically significant. Univariate and multivariable logistic regression analyses were performed for the association of EAT thickness with the bradyarrhythmia requiring PM implantation. For logistic regression analysis, related factors (age, gender, body mass index, hypertension, hyperlipidemia, diabetes, history of heart failure [HF], left ventricular ejection fraction, left atrial diameter [LAD], estimated glomerular filtration rate [eGFR], and β‐blocker) were included. To adjust for bias due to the effect of potential confounders, a propensity score matching analysis was also performed to match the patients in the PM group and control in a ratio of 1:1. The baseline characteristics (age, gender, hypertension, HF, LAD, and eGFR) were incorporated into the analysis. This analysis procedure was performed with JMP software version 11 (SAS Institute Japan), using a fixed caliper width of 0.05.

## 3 Results

### 3.1 Patient Characteristics

We finally enrolled 103 patients in the PM group and 105 in the control. The patient characteristics are shown in Table 1. There were significant differences among the age (PM group 81 ± 8 years vs. control 74 ± 6 years, *p* < 0.0001), hypertension (PM group 78[75.7%] vs. control 55[52.4%], *p* = 0.0004), history of HF (PM group 28[27.2%] vs. control 4[3.8%], *p* < 0.0001), LAD (PM group 40.6 ± 5.9 mm vs. control 35.2 ± 5.3 mm, *p* < 0.0001), and eGFR (PM group 51.6 ± 18.8 mL/min/1.73 m^2^ vs. 64.3 ± 16.4 mL/min/1.73 m^2^, *p* < 0.0001). Table 2 shows the primary diseases caused by the PM implantation and PSVT types in the control. There were 38 (36.9%) patients with sick sinus syndrome (SSS) and 65 (63.1%) with advanced AVB in the PM group. There were 20 (19.0%) patients with atrioventricular reentrant tachycardia (AVRT) and 85 (81.0%) with atrioventricular nodal reentrant tachycardia (AVNRT) in the control.

**TABLE 1:** Characteristics of the patients in the PM group and control.

No. of patients	PM group	Control	*p*
	103	105	
Age (years)	81 ± 8	74 ± 6	< 0.0001
Men, *n* (%)	55 (53.4)	50 (47.6)	0.4045
BMI (kg/m^2^)	23.6 ± 3.3	23.2 ± 3.5	0.4500
Hypertension, *n* (%)	78 (75.7)	55 (52.4)	0.0004
Hyperlipidemia, *n* (%)	55 (53.4)	48 (45.7)	0.2676
Diabetes, *n* (%)	29 (28.2)	22 (21.0)	0.2268
Heart failure, *n* (%)	28 (27.2)	4 (3.8)	< 0.0001
LVEF (%)	64.2 ± 10.3	66.5 ± 6.4	0.0544
LAD (mm)	40.5 ± 5.7	36.1 ± 5.5	< 0.0001
eGFR (mL/min/1.73 m^2^)	51.6 ± 18.8	64.3 ± 16.4	< 0.0001
β‐blocker, *n* (%)	17 (16.5)	9 (8.6)	0.0816

Abbreviations: BMI, body mass index; eGFR, estimated glomerular filtration rate; LAD, left atrial diameter; LVEF, left ventricular ejection fraction; PM, pacemaker.

**TABLE 2:** Primary disease in the PM group and the PSVT types in the control.

PM group		
	SSS	Advanced AVB
No. of patients, *n* (%)	38 (36.9%)	65 (63.1%)

Control (PSVT)		
	AVRT	AVNRT
No. of patients, *n* (%)	20 (19.0%)	85 (81.0%)

Abbreviations: AVB, atrioventricular block; AVNRT, atrioventricular nodal reentrant tachycardia; AVRT, atrioventricular reentrant tachycardia; PM, pacemaker; PSVT, paroxysmal supraventricular tachycardia; SSS, sick sinus syndrome.

### 3.2 EAT Thickness

**FIGURE 3:**
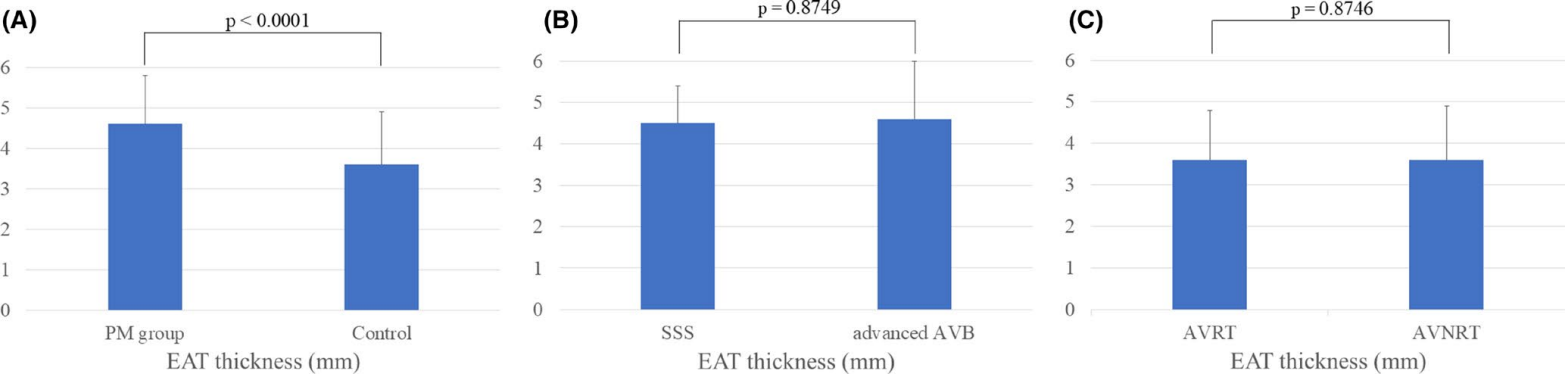
The EAT thickness in the PM group and control (A). The EAT thickness in the SSS group and advanced AVB group (B). The EAT thickness in the AVRT group and AVNRT group (C).

The EAT was significantly thicker in the PM group than control (PM group 4.6 ± 1.2 mm vs. control 3.6 ± 1.3 mm, *p* < 0.0001) (Figure 3A). We performed the multivariate analysis and revealed that age, gender, HF, LAD, and EAT thickness were independently associated with bradyarrhythmia requiring PM implantation (EAT thickness, odds ratio; 2.084, *p* < 0.0001) (Table 3). On the other hand, in the PM group, there was no significant difference in the EAT thickness between the patients with SSS and those with advanced AVB (SSS 4.5 ± 0.9 mm vs. advanced AVB 4.6 ± 1.4 mm, *p* = 0.8749) (Figure 3B). We further conducted subgroup analyses by age (Group 1: aged 65–74 years, Group 2: aged 75–84 years, Group 3: aged > 85 years) (Data S1). In all age groups, the PM group showed significantly greater EAT thickness than the control (Group 1, PM group 4.6 ± 1.8 mm vs. control 3.6 ± 1.3 mm, *p* = 0.0015, Group 2, PM group 4.6 ± 1.2 mm vs. control 3.7 ± 1.2 mm, *p* = 0.0002, Group 3, PM group 4.5 ± 1.3 mm vs. control 3.2 ± 1.6 mm, *p* = 0.0109). In the control, there was also no significant difference between the patients with AVRT and those with AVNRT (AVRT 3.6 ± 1.2 mm vs. AVNRT 3.6 ± 1.3 mm, *p* = 0.8746) (Figure 3C).

**TABLE 3:** Univariate and multivariate analysis of the related factors for bradyarrhythmia requiring PM implantation.

	Univariate			Multivariate		
	OR	95% CI	*p*	OR	95% CI	*p*
Age	1.144	1.097–1.198	< 0.0001	1.123	1.061–1.194	< 0.0001
Gender (Men)	1.260	0.732–2.178	0.4045	2.948	1.331–6.895	0.0072
BMI (kg/m^2^)	1.032	0.951–1.121	0.4472	0.886	0.776–1.008	0.0656
Hypertension	2.836	1.583–5.181	0.0004	2.207	0.978–5.101	0.0567
Hyperlipidemia	1.361	0.790–2.354	0.2676	1.746	0.815–3.805	0.1519
Diabetes	1.479	0.785–2.817	0.2268	1.066	0.425–2.681	0.8911
Heart failure	9.427	3.515–32.843	< 0.001	4.6	1.305–19.737	0.0166
LVEF (%)	0.967	0.933–1.000	0.0505	1.044	0.987–1.104	0.1342
LAD (mm)	1.155	1.094–1.225	< 0.0001	1.15	1.062–1.257	0.0005
eGFR (mL/min/1.73 m^2^)	0.961	0.944–0.976	< 0.0001	0.986	0.963–1.009	0.2303
β‐blocker	2.109	0.911–5.181	0.0816	1.234	0.375–4.173	0.7284
EAT thickness (mm)	1.846	1.457–2.391	< 0.0001	2.084	1.514–2.974	< 0.0001

Abbreviations: BMI, body mass index; CI, confidence interval; EAT, epicardial adipose tissue; eGFR, estimated glomerular filtration rate; LAD, left atrial diameter; LVEF, left ventricular ejection fraction; OR, odds ratio; PM, pacemaker.

### 3.3 EAT Thickness After Propensity Score Matching

**FIGURE 4:**
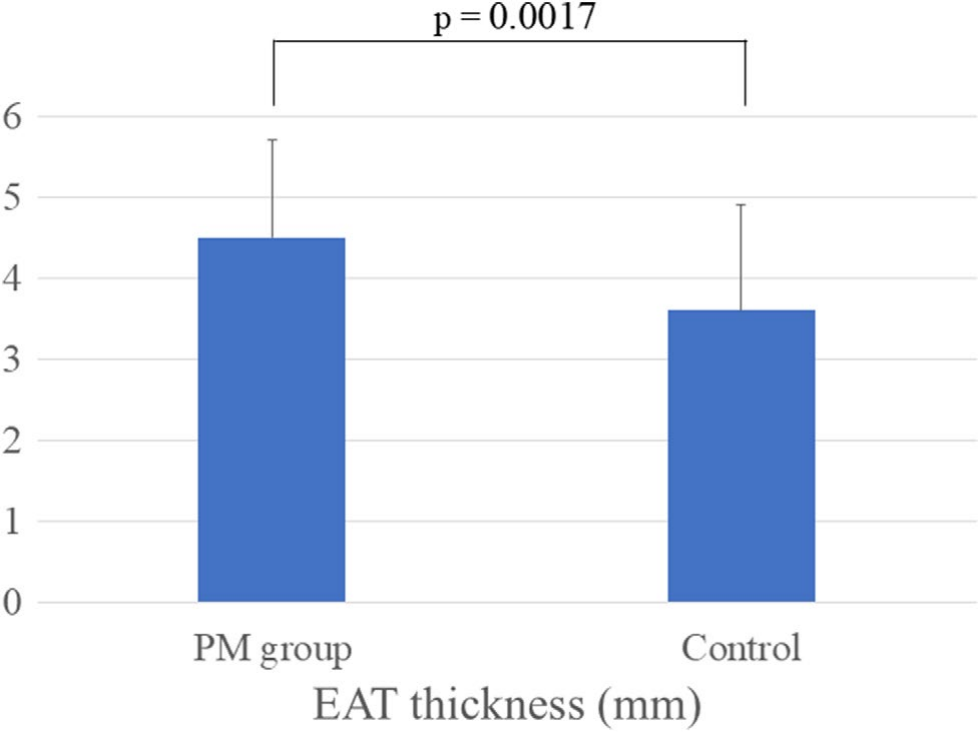
The EAT thickness in the PM group and control after propensity score matching.

A total of 72 patients (PM group and control, 1:1) were included in the analysis after propensity score matching. Table 4 shows the patient characteristics, and there were no significant differences among all items. The EAT was also significantly thicker in the PM group than in the control (PM group 4.5 ± 1.2 mm vs. Control 3.6 ± 1.3 mm, *p* = 0.0017) (Figure 4) after matching.

**TABLE 4:** Characteristics of the patients in the PM group and control after propensity score matching.

No. of patients	PM group	Control	*p*
	36	36	
Age (years)	76 ± 8	77 ± 6	0.8343
Men, *n* (%)	23 (63.9)	17 (47.2)	0.1537
BMI (kg/m^2^)	22.8 ± 2.7	24.0 ± 3.9	0.1484
Hypertension, *n* (%)	25 (69.4)	28 (77.8)	0.4217
Hyperlipidemia, *n* (%)	18 (50.0)	18 (50.0)	1.0000
Diabetes, *n* (%)	9 (25.0)	8 (22.2)	0.7813
Heart failure, *n* (%)	4 (11.1)	3 (8.33)	0.6903
LVEF (%)	65.9 ± 8.7	65.0 ± 6.7	0.6295
LAD (mm)	37.6 ± 4.8	38.2 ± 4.4	0.6057
eGFR (mL/min/1.73 m^2^)	57.6 ± 20.2	59.3 ± 17.9	0.7040
β‐blocker, *n* (%)	3 (8.3)	3 (8.3)	1.0000

Abbreviations: BMI, body mass index; eGFR, estimated glomerular filtration rate; LAD, left atrial diameter; LVEF, left ventricular ejection fraction; PM, pacemaker.

## 4 Discussion

In this study, we revealed that the EAT thickness was independently associated with bradyarrhythmia requiring permanent PM implantation in elderly people. The EAT was also significantly thicker in the PM group than in the control after adjusting for age, hypertension, history of HF, LAD, and eGFR. Aging is one of the most significant risk factors for bradyarrhythmia, but not all elderly people need PMs, and other risk factors possibly exist. It has been reported that there is a modest correlation between the EAT volume on cardiac magnetic resonance imaging (MRI) and the EAT thickness on echocardiography [12]. Therefore, cases with a thicker EAT in the anterior right ventricle are likely to have a thicker EAT around the SN and AVN as well. It has also been reported that three of 14 cases treated with PMs for SN dysfunction exhibited AVN dysfunction, and two of these 3 cases had excessive fatty infiltration around the atrio‐nodal junction area and into the atrophic AVN itself in the histopathological findings [13]. A recent report showed the association between bradyarrhythmias (SSS and AVB) and EAT [14]. The report indicated that SSS and AVB are each associated with increased EAT volume, with third‐degree AVB showing a greater volume. Histological examination of the left atrial appendage after resection has reported that areas of fibrosis are found in myocardial cells adjacent to adipocytes [5]. A report examining the relationship between EAT and patients with atrial fibrillation who underwent catheter ablation indicated that the EAT volume was associated with left atrial conduction disturbance [15]. Excessive amounts of EAT may directly infiltrate around the SN and AVN or fibrosis may develop due to inflammatory cytokines, which may affect SN and AVN function. EAT includes the autonomic plexus and promotes unbalanced autonomic remodeling [16]. It has also been reported that a highly significant correlation exists between the EAT thickness and cardiac sympathetic denervation assessed by cardiac ^123^I‐MIBG [17]. Cardiac sympathetic denervation may cause bradyarrhythmia like intermittent SSS and advanced AVB associated with the autonomic nervous system. Some papers have reported the association between EAT and coronary artery disease [18, 19, 20]. An increasing amount of EAT is linked to an augmented inflammatory reaction, leading to the development and progression of coronary atherosclerosis [21]. Atherosclerosis in the SN artery or AVN artery may influence the SN or AVN function due to ischemia of the SN or AVN. Further analyses are needed to elucidate the mechanism by which EAT promotes bradyarrhythmia.

## 5 Limitations

There were some limitations to our study.

First, this study was a retrospective observational study in a single center. Therefore, the sample size was small, and further prospective studies are needed to confirm the accuracy of our results. Second, we used the transthoracic echocardiography–derived EAT thickness due to the broad accessibility because some patients with bradyarrhythmia required PMs for temporary pacing or had hemodynamic compromise and could not undergo computed tomography or MRI. Although the EAT around the SN and AVN has not been directly measured, the EAT thickness correlates with the EAT volume around the heart, and the EAT thickness on the anterior side of the right ventricle also possibly correlates with the EAT thickness around the SN and AVN.

## 6 Conclusions

The EAT thickness is associated with bradyarrhythmia requiring permanent PM implantation and may become a risk factor for SN and AVN dysfunction.

## Funding

The authors have nothing to report.

## Ethics Statement

This study was approved by the institutional ethics committee of Hiroshima Prefectural Hospital.

## Conflicts of Interest

The authors declare no conflicts of interest.

## Supporting information


**Data S1:** joa370340‐sup‐0001‐Supinfo.docx.

## Data Availability

The data that support the findings of this study are available on request from the corresponding author. The data are not publicly available due to privacy or ethical restrictions.
