# Supplementary material for: Association Between Bradyarrhythmia Requiring Permanent Pacemaker Implantation and Epicardial Adipose Tissue in Elderly Patients
Source: J Arrhythm. 2026 Apr 16;42(2):e70340. doi: 10.1002/joa3.70340 (PMC13084190; doi:10.1002/joa3.70340)

Supplementary Material

Subgroup Analyses by Age

|  | PM group | Control |
| --- | --- | --- |
| No. of patients | 103 | 105 |
|  |  |  |
| Group1: aged 65 to 74 years, n (%) | 23 (22.3) | 56 (53.3) |
|  |  |  |
| Group2: aged 75 to 84 years, n (%) | 37 (35.9) | 40 (38.1) |
|  |  |  |
| Group3: aged >85 years, n (%) | 43 (41.8) | 9 (8.6) |

Group1: (PM group vs. Control)

p = 0.0015


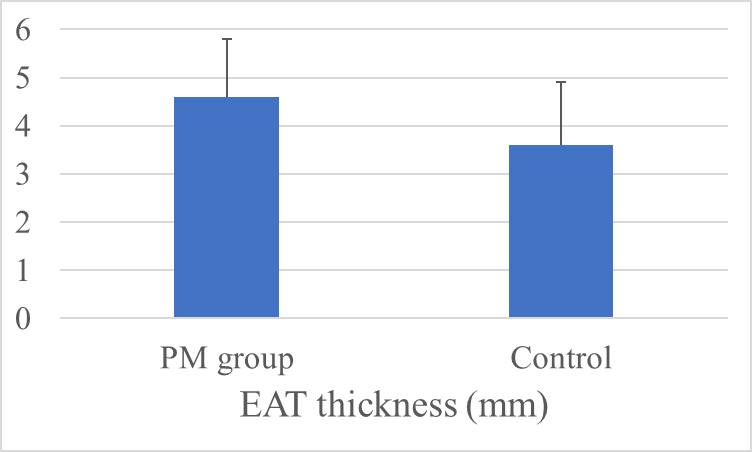


Group2: (PM group vs. Control)

p = 0.002


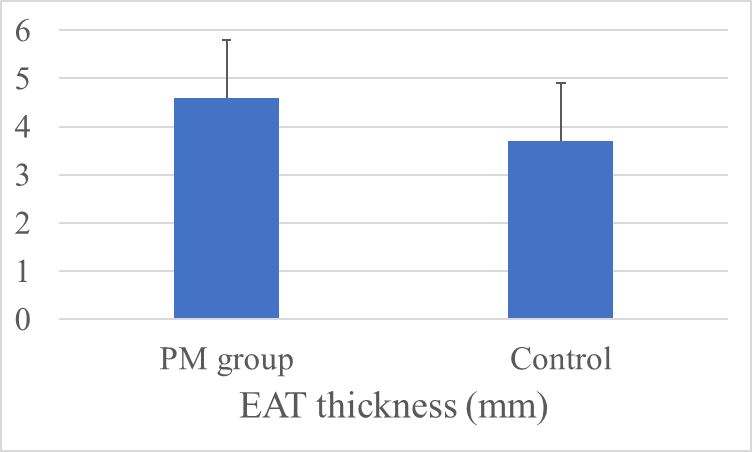


Group3: (PM group vs. Control)

p = 0.0109


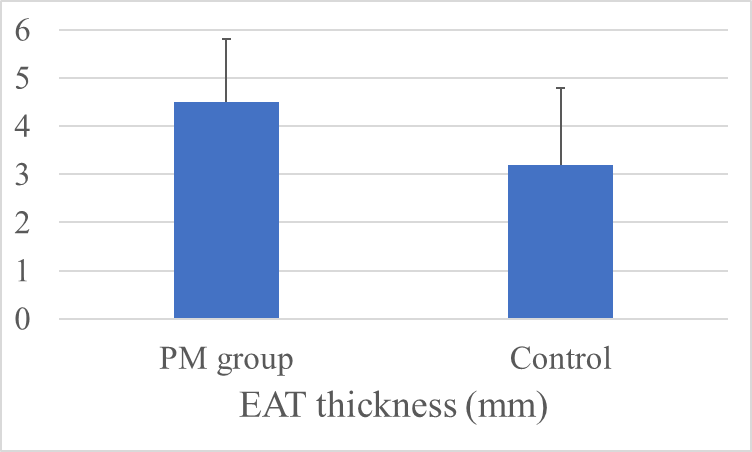

Supplement: Supplementary file 1 — Data S1: joa370340‐sup‐0001‐Supinfo.docx. [file JOA3-42-e70340-s001.docx]
